# Lateral Extra‐articular Tenodesis Utilizing Modified Ellison Technique With Knotless Anchor Fixation

**DOI:** 10.1002/atn2.70062

**Published:** 2026-06-15

**Authors:** Joseph M. Sliepka, Natalie K. Kucirek, Alan L. Zhang

**Affiliations:** ^1^ Department of Orthopaedic Surgery University of California‐San Francisco San Francisco California U.S.A.

## Abstract

Lateral extra‐articular tenodesis (LET) to augment anterior cruciate ligament reconstruction significantly reduces graft failure rates. Although there are many techniques for lateral extra‐articular tenodesis, the modified Ellison technique provides numerous advantages as a distally fixed construct, including dynamic rotational stability, reducing the risk of lateral compartment overload, and eliminating femoral tunnel convergence. The addition of knotless anchor fixation provides a reproducible and efficient method for providing extra‐articular stability to anterior cruciate ligament reconstruction without the need to alter postoperative rehabilitation protocols. We describe our preferred lateral extra‐articular tenodesis technique utilizing the modified Ellison approach with both an all‐suture knotless anchor and a hardbody knotless anchor to provide stable fixation while minimizing additional operative time during anterior cruciate ligament reconstruction.

## Graphical abstract

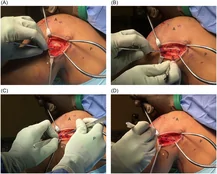

**VIDEO 1:** In this video, we will present a modified Ellison technique for lateral extra‐articular tenodesis using knotless suture anchors. Gerdy's tubercle, the fibular head, and the medial epicondyle are marked out as anatomic landmarks. An 8 cm longitudinal skin incision is made in line with the distal femur, ending distally between Gerdy's tubercle and the fibular head. Dissection is carried down to the iliotibial (IT) band, exposing its anterior and posterior borders. A 10 mm wide strip of IT band is marked out, starting from Gerdy's tubercle and traveling proximally about 7 cm. The strip is then cut anteriorly and posteriorly and released distally from Gerdy's tubercle. Care is taken to release the entire depth of the IT band while staying above joint capsule. The proximal extent is left attached to IT band, unlike in a modified Lemaire technique. The strip is then tagged distally with number 2 orthocord using a Krakow technique. The lateral collateral ligament (LCL) is then identified and isolated. Placing a varus stress on the knee can help define its borders. Blunt dissection is used to isolate the LCL and a hemostat is passed deep to the LCL from anterior to posterior. The tagging suture of the IT band slip is then grasped with the hemostat and the slip is passed deep to the LCL from posterior to anterior. An Iconix #2 knotless anchor containing 1 repair strand and 1 looped shuttle strand is inserted 1 cm proximal and 5 mm posterior to Gerdy's tubercle. A 2.3 mm drill bit is used to drill a pilot hole with a depth of 21.5 mm and the anchor is placed. The IT band slip is placed between the shuttle strand and the repair strand of the Iconix #2 knotless anchor as depicted in this video and in the following still shots. The repair strand is then fed through the shuttle strand and shuttled into the anchor—fixing the IT band slip in the center. The orthocord sutures tagging the IT band slip are then passed through a Knotilus 2.9 mm PEEK anchor and held tight as the anchor is placed at Gerdy's tubercle, providing backup fixation. The tagging suture can be tensioned both before anchor placement and again just before the anchor thread engages in bone. In this image, the probe shows the position of the Iconix anchor, while the scissors show the position of the Knotilus anchor. The wound is then irrigated and the IT band closed. Video content can be viewed at https://doi.org/10.1002/atn2.70062.

Lateral extra‐articular tenodesis (LET) as an adjunct to anterior cruciate ligament (ACL) reconstruction has enjoyed renewed popularity in recent years.^1^, ^2^, ^3^ LET procedures typically use a strip of the iliotibial (IT) band to reinforce the lateral aspect of the knee, helping provide rotational stability.^4^ Recent studies have shown that the addition of LET to ACL reconstruction can help reduce rerupture rates without significantly increasing complications.^5^, ^6^, ^7^, ^8^

Many techniques exist to help provide lateral extra‐articular stability. The modified Lemaire technique harvests a strip of IT band while leaving the distal insertion intact, passing it deep to the lateral collateral ligament (LCL), and then reattaching to the lateral femoral condyle.^9^ The Arnold‐Coker modification of the McIntosh technique is similar to the Lemaire in that it also harvests a strip of the IT band leaving its distal insertion intact; however, it is then looped around distally and attached to Gerdy's tubercle.^10^ The modified Ellison technique involves harvesting a strip of the IT band, leaving it attached proximally, and elevating it off its distal insertion at Gerdy's tubercle. It is then passed deep to LCL and reattached just anterior to Gerdy's tubercle.^11^, ^12^, ^13^, ^14^

Studies directly comparing outcomes between the different methods for LET are sparse, and no single technique has shown superiority compared with others. Benefits of adding LET to ACL reconstruction include reducing graft failure rates, improving rotational stability, and reducing postoperative pivot shift.^6^, ^7^, ^8^, ^15^ The risks of additional LET include increasing early postoperative pain and stiffness, wound complication and infection risks, and lateral compartment overconstraint, although this has not been fully shown in the literature.^6^, ^15^, ^16^ Given the clinical benefits that LET provide without clear superiority of a single technique, it is valuable to describe technique variations that may be advantageous. We describe our preferred LET technique utilizing the modified Ellison approach with 2 knotless anchors to provide stable fixation while minimizing additional operative time during ACL reconstruction.

## SURGICAL TECHNIQUE

**FIGURE 1:**
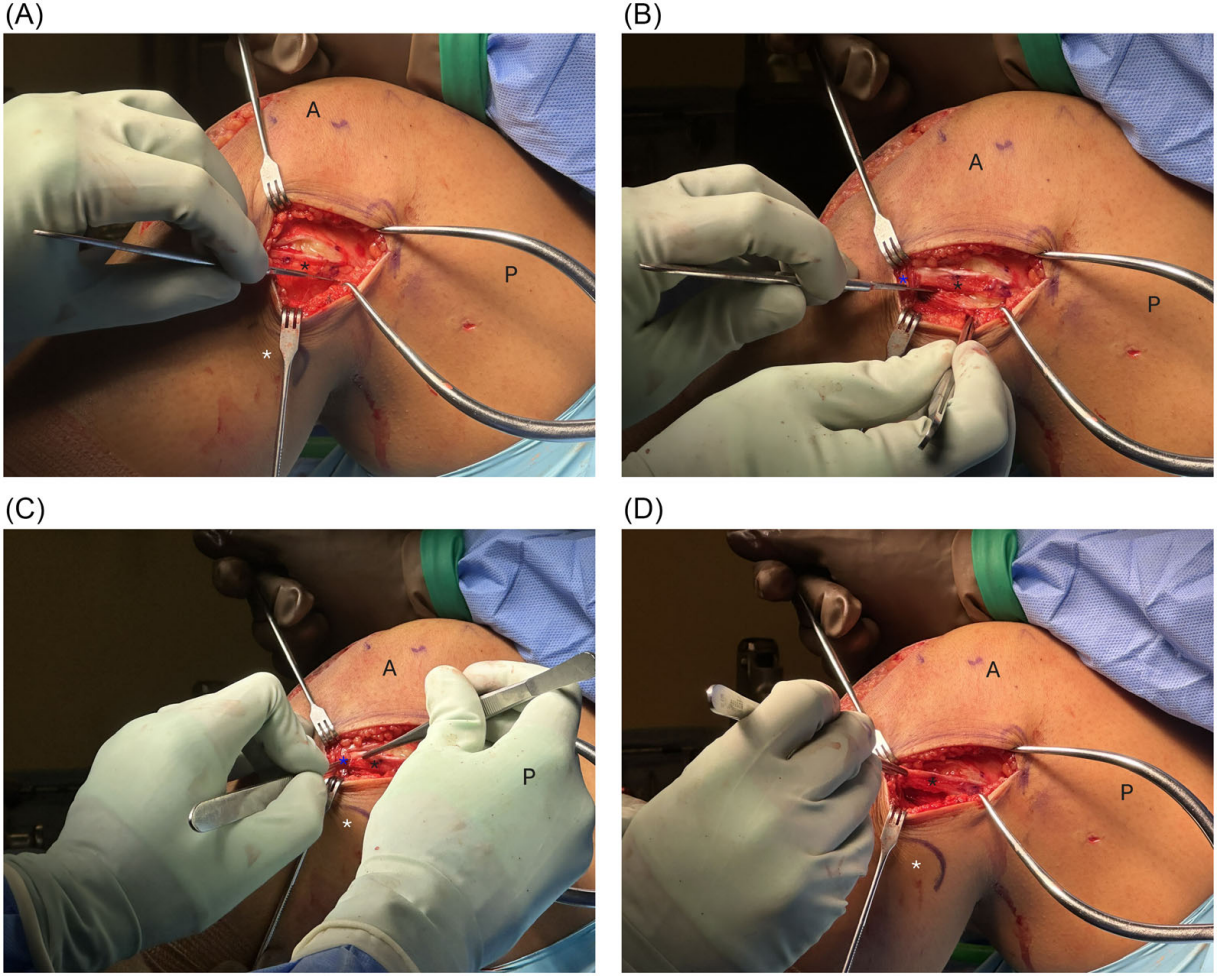
Intraoperative photos during IT band harvest of the left knee in the supine position, with the distal aspect of the extremity on the left, show isolating the (A) anterior and (B) posterior borders of a 10 mm width strip of IT band. (C) The IT band is then released off its insertion at Gerdy's tubercle. (D) A 7 cm length strip of IT band is harvested, leaving the capsule underneath protected. The black, white, and blue asterisks demarcate the central slip of the IT band, the fibular head, and Gerdy's tubercle, respectively. For reference, the letters P and A represent proximal and anterior. (IT, iliotibial.)

**FIGURE 2:**
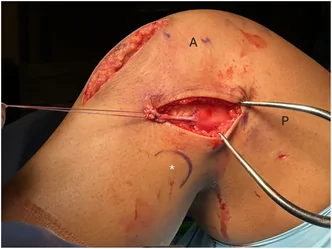
Intraoperative photo during IT band harvest of the left knee in the supine position, with the distal aspect of the extremity on the left. Once the 10 mm strip is isolated, it is tagged with suture utilizing Krakow technique in locking fashion. The black and white asterisks demarcate the IT band slip and the fibular head, respectively. For reference, the letters P and A represent proximal and anterior. (IT, iliotibial.)

When performing LET utilizing the modified Ellison approach with knotless anchors (Video 1), the ACL reconstruction can first be fully completed with the graft fixed in both the femoral and tibial tunnels prior to starting the LET. The patient is positioned supine with the knee flexed to 90°, held stable by a foot positioner at the foot of the bed and a post preventing external rotation of the hip. A tourniquet is not needed for this procedure. A 7 cm longitudinal incision is made over the lateral distal femur in line with the femur ending distally between Gerdy's tubercle and the fibular head. Sharp dissection is carried down to the IT band. A 10 mm strip of the central portion of the IT band is isolated and released off its distal insertion on Gerdy's tubercle, leaving the anterior and posterior borders of IT band intact (Figure 1A‐D). Once the strip is isolated, it is tagged with suture (#2 orthocord; Depuy Synthes, Warsaw, IN) utilizing the Krakow technique in locking fashion (Figure 2). This strip of IT band can be extended proximally as needed to provide enough excursion to complete the procedure, usually around 7 cm in length.

**FIGURE 3:**
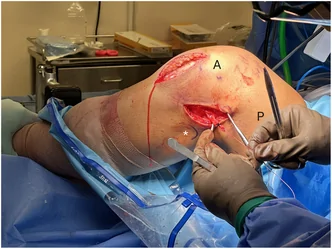
Intraoperative photo during lateral extra‐articular tenodesis of the left knee in the supine position, with the distal aspect of the extremity on the left. Placing a varus moment on the knee, such as in a figure 4 position, can help with locating the LCL prior to IT band passage. The black and white asterisks demarcate the IT band slip and the fibular head, respectively. For reference, the letters P and A represent proximal and anterior. (IT, iliotibial; LCL, lateral collateral ligament.)

**FIGURE 4:**
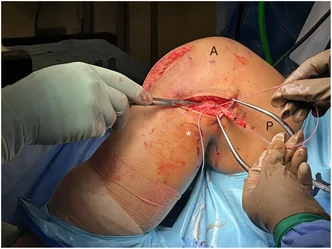
Intraoperative photo during lateral extra‐articular tenodesis of the left knee in the supine position, with the distal aspect of the extremity on the left. After the LCL is isolated, a blunt instrument such as a hemostat is passed deep to the LCL from anterior to posterior to allow for easy graft passage. The black, white, and blue asterisks demarcate the IT band slip, the fibular head, and the LCL, respectively. For reference, the letters P and A represent proximal and anterior. (IT, iliotibial; LCL, lateral collateral ligament.)

**FIGURE 5:**
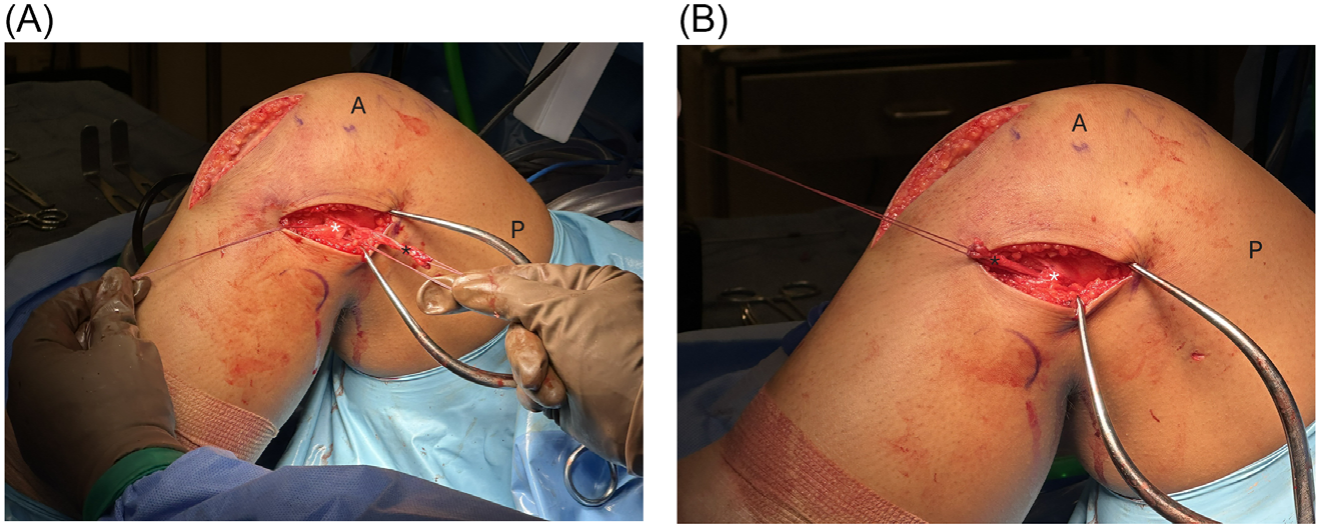
Intraoperative photos of IT band passage of the left knee in the supine position, with the distal aspect of the extremity on the left. (A) The tagging sutures are first shuttled deep to the LCL. (B) The tagging sutures are then used to help shuttle the IT band strip deep to LCL from posterior to anterior. The black and white asterisks demarcate the IT band strip and LCL, respectively. For reference, the letters P and A represent proximal and anterior. (IT, iliotibial; LCL, lateral collateral ligament.)

The LCL is then isolated. Placing a varus moment on the knee, such as in a figure 4 position, can help with locating the LCL (Figure 3). Blunt dissection on either side of the LCL isolates the ligament to assist in ease of suture passage. A blunt instrument such as a hemostat is passed deep to the LCL from anterior to posterior (Figure 4). This is used to help shuttle the IT band tagging suture deep to LCL, which then can be pulled to pass the IT band strip deep to LCL from posterior to anterior (Figure 5A,B).

**FIGURE 6:**
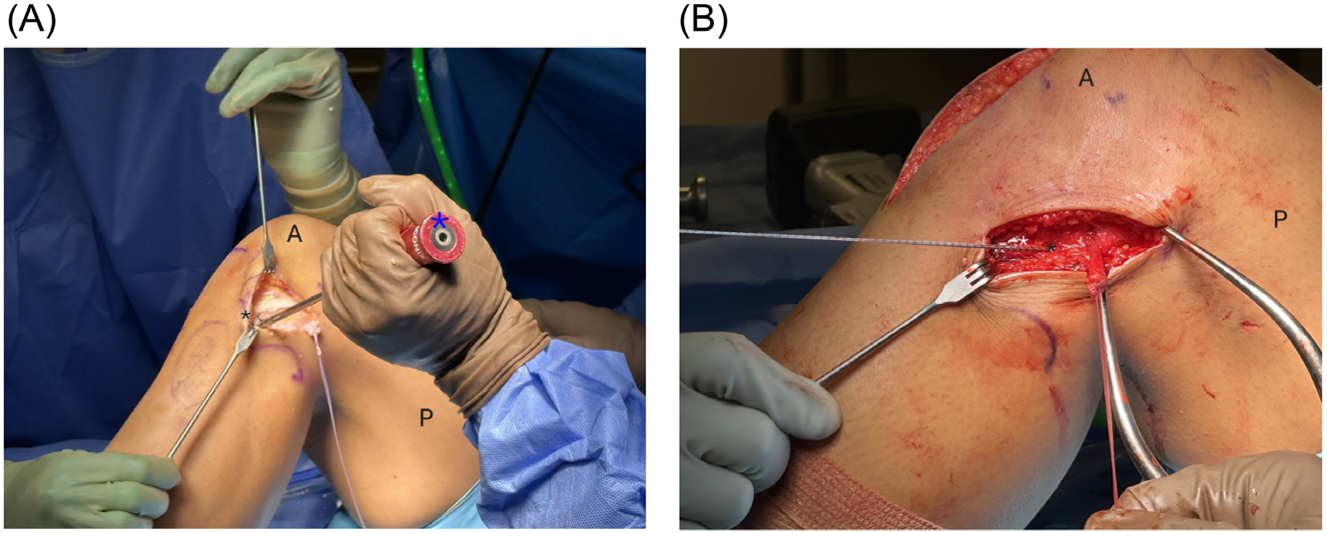
Intraoperative photos of first knotless anchor placement of the left knee in supine position, with the distal aspect of the extremity on the left. (A) A drill is first used to create a pilot hole 1cm proximal and 5 mm posterior to Gerdy's tubercle, after which the anchor is placed. (B) This anchor contains one repair strand and one looped shuttle strand. The black, white, and blue asterisks represent Gerdy's tubercle, the knotless 2.3 mm all suture anchor (Iconix 2 knotless; Stryker, Kalamazoo, MI), and the IR band slip, respectively. For reference, the letters P and A represent proximal and anterior.

**FIGURE 7:**
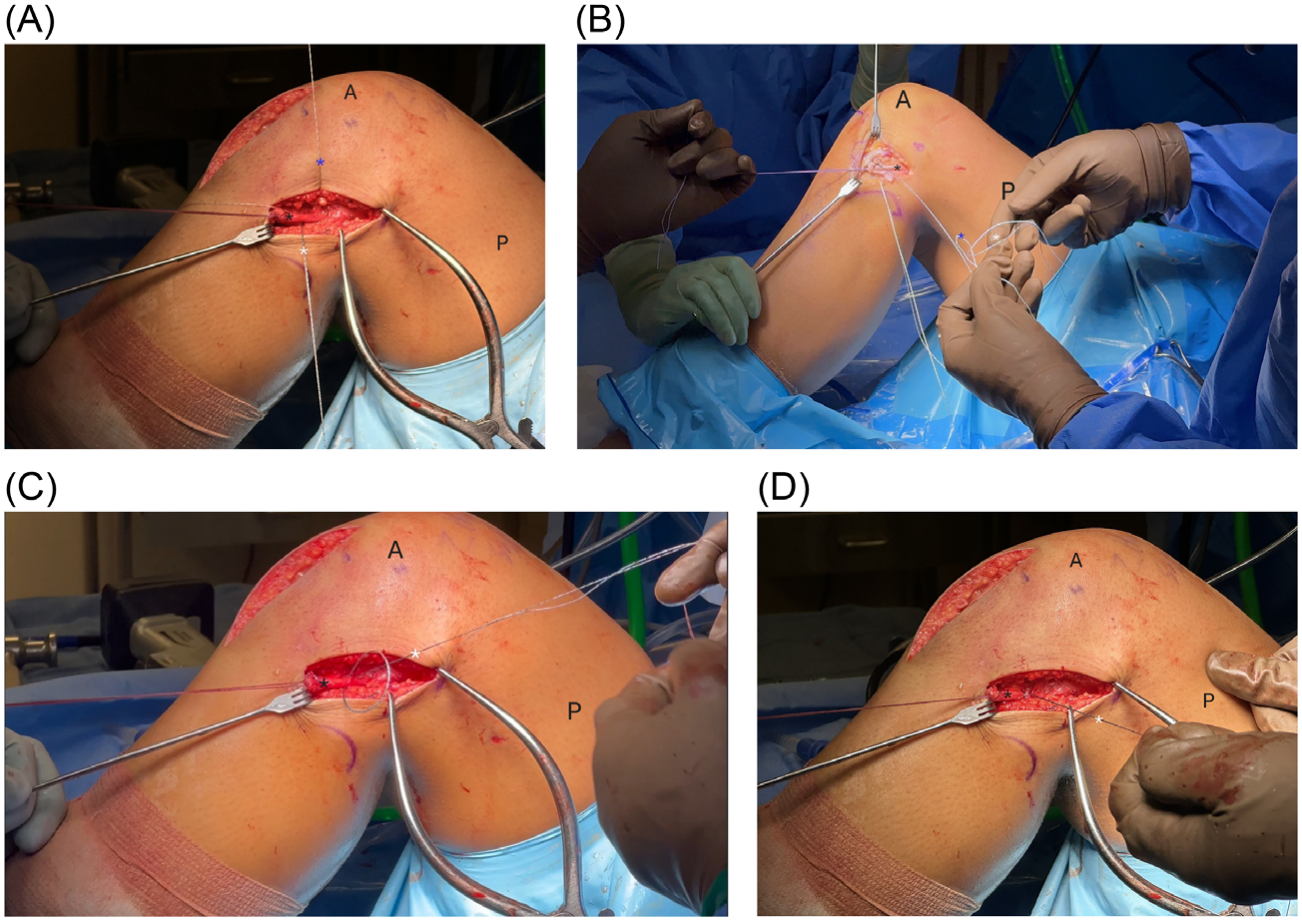
Intraoperative photos of first knotless anchor placement of the left knee in supine position, with the distal aspect of the extremity on the left. (A) Once the anchor is secured, the IT band slip is then placed between the looped shuttle strand and the repair strand. (B) The repair strand is then passed through the loop of the shuttle strand until it reaches the demarcation of suture color change, where it is folded and pinched to the shuttle strand loop. (C) The shuttle strand is then pulled, passing the repair strand around the graft. (D) Slight tension is then applied to the repair strand, securing the graft. The black, white, and blue asterisks represent the IT band slip, the repair strand, and the looped shuttle strand, respectively. For reference, the letters P and A represent proximal and anterior. (IT, iliotibial.)

**FIGURE 8:**
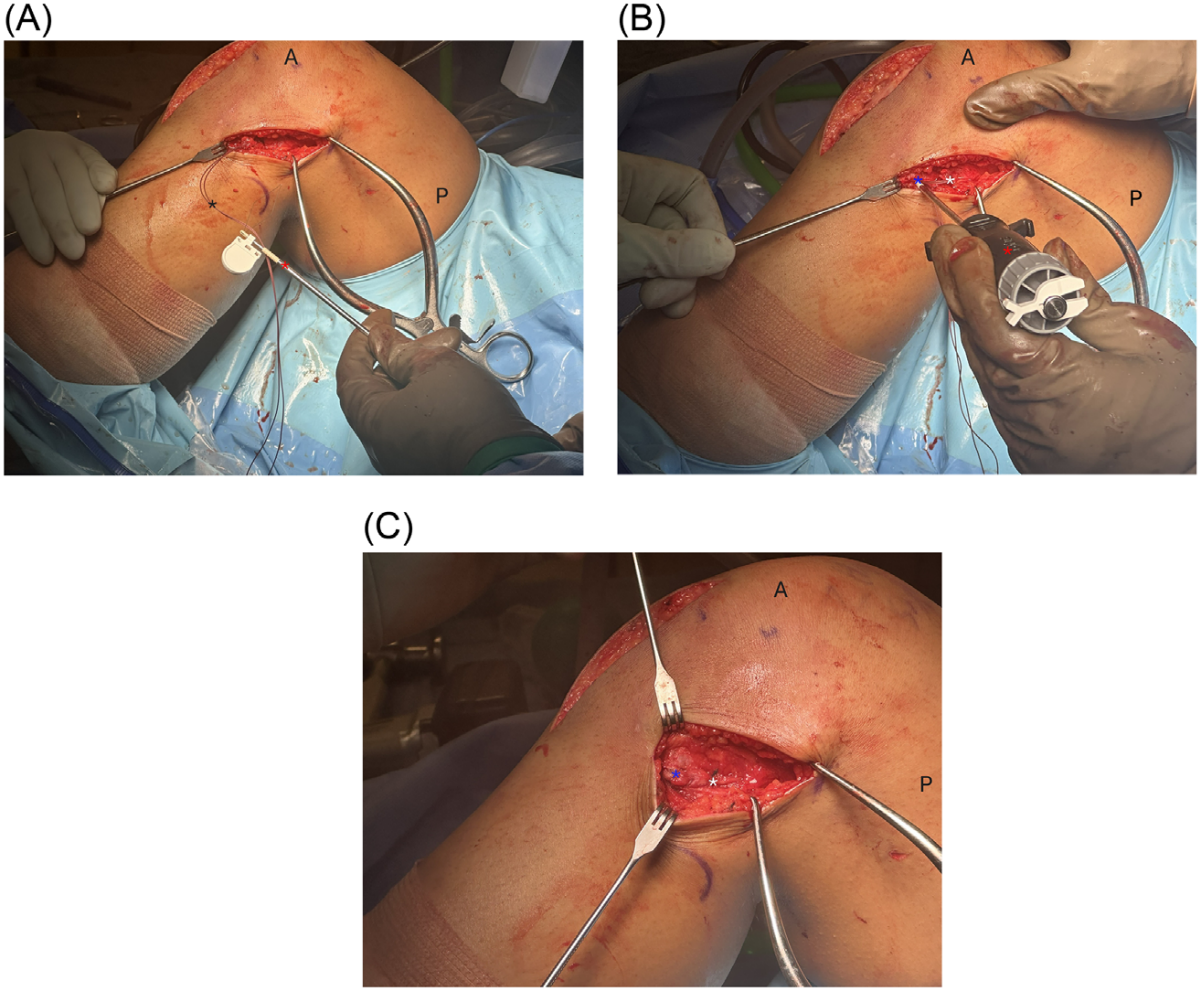
Intraoperative photos of second knotless anchor placement of the left knee in supine position, with the distal aspect of the extremity on the left. (A) The tagging sutures of the IT band strip are then loaded through the eyelet of the knotless anchor. (B) A drill is then used to create a pilot hole at Gerdy's tubercle, after which the anchor is placed. (C) The sutures are then held in tension as the anchor is secured, and residual suture is cut. The black, white, and blue asterisks represent the IT band tagging sutures, the location of the first anchor, and Gerdy's tubercle, respectively. The red asterisk represents the 2.9 mm PEEK knotless anchor (Knotilus+; Stryker, Kalamazoo, MI). For reference, the letters P and A represent proximal and anterior. (IT, iliotibial.)

**FIGURE 9:**
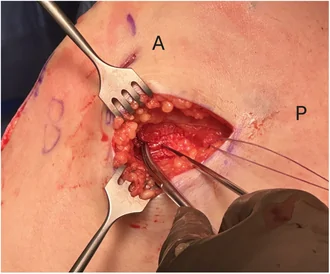
Intraoperative photo during lateral extra‐articular tenodesis of the left knee in supine position showing the location of placement of each anchor, with the distal aspect of the extremity on the left. The first anchor is placed just proximal to Gerdy's tubercle, denoted by the probe (right), and the second anchor is placed at Gerdy's tubercle, denoted by the scissors (left). For reference, the letters P and A represent proximal and anterior. For reference, the letters P and A represent proximal and anterior.

**FIGURE 10:**
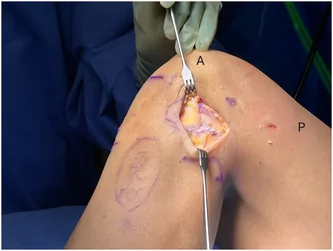
Intraoperative photo of the IT band closure over LET construct of the left knee in supine position, with minimal tension and bulk added. The distal aspect of the extremity is on the left. For reference, the letters P and A represent proximal and anterior. For reference, the letters P and A represent proximal and anterior. (IT, iliotibial; LET, lateral extra‐articular tenodesis.)

The knotless 2.3 mm all suture anchor (Iconix 2 knotless; Stryker, Kalamazoo, MI) is then placed approximately 1 cm proximal and 5 mm posterior to Gerdy's tubercle (Figure 6A,B). A 2.3 mm drill is first used to create a pilot hole to a depth of 21.5 mm after which the anchor is placed, which contains 1 repair strand and 1 looped shuttle strand. The suture strands are pulled upward slightly to set the anchor. The IT band slip is then placed over the anchor with the repair strand and the shuttle strand split on either side of it. The repair strand is then shuttled into the anchor by the shuttle strand. In this step, care is taken to pass the repair strand through the loop of the shuttle strand until it reaches the demarcation of suture color change. At the demarcation, the repair strand is folded and pinched to the shuttle strand loop. Slight tension is then applied to the IT band strip as the repair strand is passed and tightened down to the anchor by pulling the shuttle strand until the tail of the repair strand is fully through the anchor. The tail of the repair strand is then pulled in short pulsating fashion until there is a positive stop to tighten the repair strand to the anchor with the IT band in the center (Figure 7A‐D). With the knee still at 90° of flexion, the tagging sutures on the IT band strip are then loaded through the eyelet of a 2.9 mm PEEK knotless anchor (Knotilus+; Stryker, Kalamazoo, MI) and passed through the anchor. A 2.9 mm drill is used to drill a pilot hole at Gerdy's tubercle and tension is held on the sutures of the IT band strip as the anchor is malleted into the pilot hole, providing stable backup fixation (Figure 8A‐C). By using this second anchor, the IT band slip does not have to be folded back on itself to prevent pull out, thereby reducing the overall bulk of the construct. The excess suture is then cut and removed. The locations of both anchors relative to each other is shown (Figure 9). After wound irrigation and achieving hemostasis, the IT band is then closed over the construct, and soft tissue and skin is closed in standard layered fashion (Figure 10). If LET is being performed with ACL reconstruction, this can be done at any point in the procedure, including after the ACL graft has been tensioned and fixed proximally and distally. This entire LET procedure can be performed and tensioned at 90° of knee flexion due to the dynamic stability provided by the modified Ellison procedure.

### Postoperative Rehabilitation

The addition of this LET technique does not change typical ACL rehabilitation protocol. Patients can weight bear as tolerated on crutches and a hinge knee brace locked in full extension for 2 weeks followed by discontinuing crutches and walking with the brace unlocked and range of motion from 0° to 90° until 4 weeks after surgery.

## DISCUSSION

LET as an adjunct to ACL reconstruction has grown in popularity as evidence for its benefit to knee stability continues to grow. There are many techniques for LET that all provide additional rotational stability to the knee when used in conjunction with ACL reconstruction. Although most of these are based on a proximally fixed construct, the modified Ellison is unique in that is fixed distally in the region of Gerdy's tubercle.^11^, ^12^, ^14^

Compared with other LET techniques, our technique provides certain advantages (Table 1). By attaching the IT band distally at a single point, it provides dynamic rather than static rotational stability. As a result, the LET does not have to be secured in any specific position of knee flexion and can be done at any point in the case. We prefer performing the LET at 90° of knee flexion for ease of positioning. By maintaining the same start and end points as the native IT band but traveling deep to LCL, this construct should tighten more in extension, where the pivot shift phenomenon occurs. In addition, the modified Ellison technique of distal fixation negates the risk of lateral compartment overconstraint. Furthermore, by not fixing the IT band to the femur, there is no concern for tunnel convergence with the ACL femoral socket. Finally, with the use of knotless anchor fixation, this technique provides an efficient way to add stability to the knee without incurring any risk of significantly increased time under anesthesia compared with other LET techniques that may require suturing of the IT band strip to knotted anchors.

**TABLE 1:** Advantages and Disadvantages of LET Utilizing Modified Ellison Technique with Knotless Suture Anchor Fixation

Advantages	Disadvantages
Knotless technique allows for simpler and faster surgery, reducing OR time and time under anesthesia	Modified Ellison technique provides a single point of fixation on the tibia, possibly leading to a less stable construct
Dynamic tensioning of modified Ellison technique allows for LET to be done at any point in ACL reconstruction	Looped fixation (without backup) could lead to graft pullout
Dynamic tensioning eliminates need for IT band to be fixed in a specific knee flexion position, or need to hold IT band at specific tension	Increased OR costs from 2 implants used in this technique
Reduces theoretical risk of lateral compartment overload	
Isolated distal fixation eliminates risk of femoral tunnel convergence when done with ACL reconstruction	
IT band slip does not have to be doubled back and fixed again, reducing overall bulk	

ACL, anterior cruciate ligament; IT, iliotibial; LET, lateral extra‐articular tenodesis; OR, operative room.

Limitations of this technique include its use of a second implant as a post that could incur increased cost per surgery. In addition, the modified Ellison technique is fixed distally compared with the modified Lemaire which is fixed both proximally and distally. Although this reduces the risk of lateral compartment overconstraint, it also increases the risk of reduced stability. Lastly, as with any LET technique, care must be taken to avoid injury to the LCL during dissection or graft passage (Table 2).

**TABLE 2:** Pearls and Pitfalls of LET Utilizing Modified Ellison Technique with Knotless Suture Anchor Fixation

Pearls	Pitfalls
Isolating the anterior and posterior borders of IT band allows for harvesting the central slip, leaving anterior and posterior fibers of IT band intact	Unlike the modified Lemaire technique, be sure to leave proximal aspect of IT band slip attached to native IT band
Tagging IT band with a secure suture technique such as a Krakow or locking stitch can reduce the risk of suture pullout, as these sutures will be passed through the second knotless anchor for final fixation	When harvesting IT band at the level of the joint, overzealous dissection can lead to capsule disruption and joint penetration
IT band tagging sutures should be placed with care, and the end of the tendon can be bulleted to help with ease of passing the graft deep to LCL	When isolating the LCL and during graft passage, care must be taken to avoid overzealous dissection and retraction to prevent injury to the LCL
Isolating LCL prior to passage of sutures and graft helps assure IT band is being passed deep to LCL	Care must be taken to not overtension the IT band when securing anchors as this could overtighten the lateral side
Secondary anchor fixation should be at the level of Gerdy's tubercle or distal, as to prevent excess slack on IT band slip when construct completed	The anchor that is placed proximal and posterior to Gerdy's tubercle must be kept distal to the joint line to avoid penetration and injury to the tibial cartilage or meniscus

IT, iliotibial; LCL, lateral collateral ligament; LET, lateral extra‐articular tenodesis.

Performing LET with the modified Ellison technique utilizing knotless anchors provides a safe and efficient way to achieve stable fixation to the knee.

## DISCLOSURES

The author (A.L.Z.) declares the following financial interests/personal relationships which may be considered as potential competing interests: A.L.Z. reports a relationship with Stryker that includes consulting or advisory; reports a relationship with DePuy Synthes Mitek Sports Medicine that includes consulting or advisory; reports a relationship with CONMED that includes consulting or advisory; reports a relationship with *Arthroscopy* that includes Associate Editor. The other authors (J.M.S., N.K.K.) declare that they have no known competing financial interests or personal relationships that could have appeared to influence the work reported in this paper.
